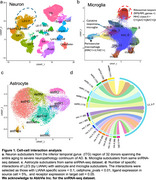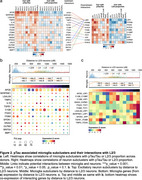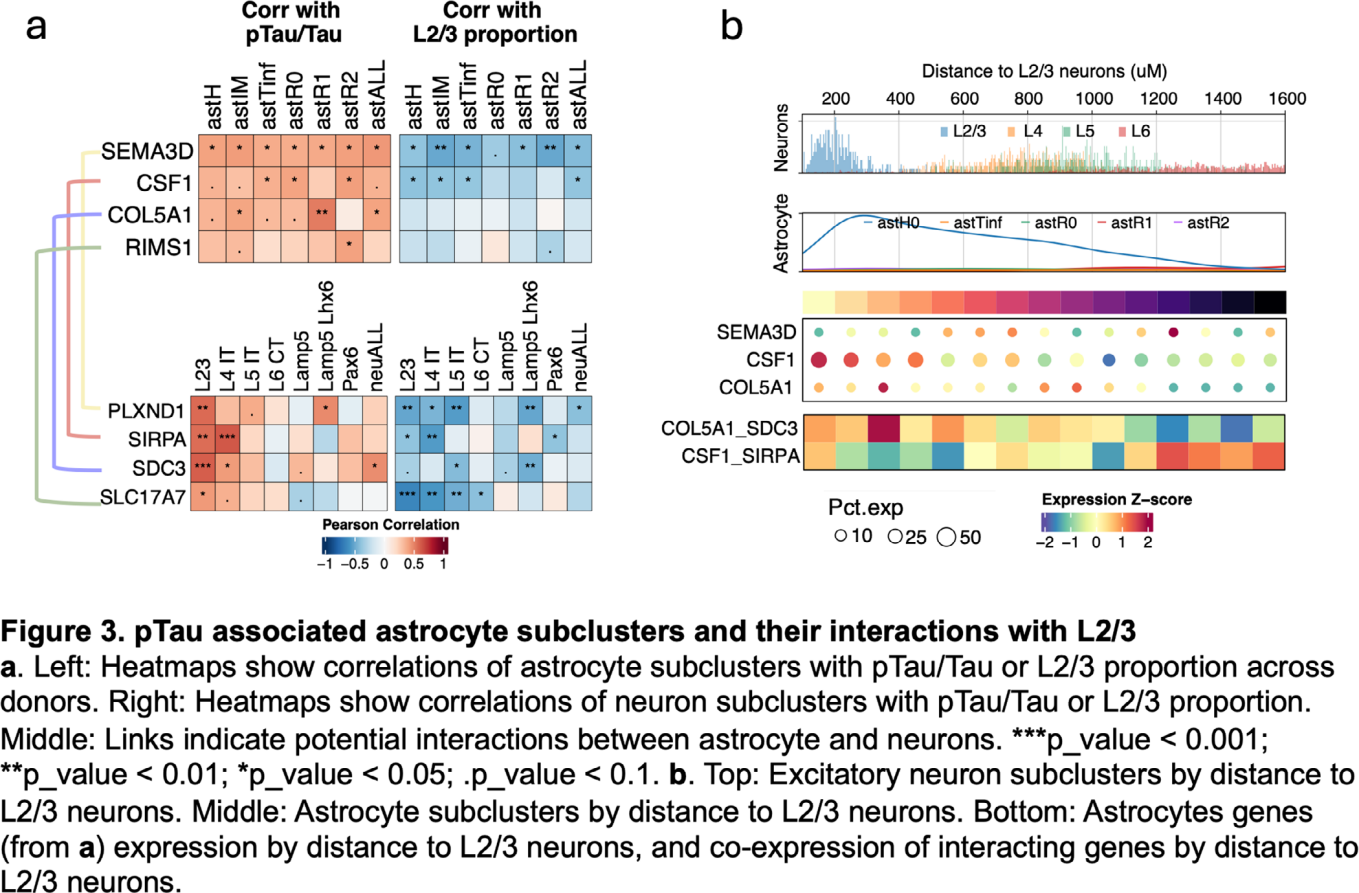# Cell‐Cell Interactions Associated with pTau Induced L2/3 Neuronal Loss in Alzheimer's Disease

**DOI:** 10.1002/alz70855_105911

**Published:** 2025-12-24

**Authors:** Huan Li, Theodore J. Zwang, Alberto Serrano‐Pozo, Bradley T. Hyman, Rachel E Bennett, Sudeshna Das

**Affiliations:** ^1^ Massachusetts General Hospital, Boston, MA, USA; ^2^ Harvard Medical School, Boston, MA, USA

## Abstract

**Background:**

Alzheimer's disease (AD) is characterized by significant neocortical layer 2/3 (L2/3) neuronal loss that correlates with the abundance of pTau and neurofibrillary tangles. While single‐nucleus studies have begun to unravel the molecular mechanisms associated with *p*‐Tau within these L2/3 neurons, the contribution of other non‐neuronal cells remains largely unknown. To address this gap, we conducted cell‐cell communication analysis using single nucleus RNA‐Seq (snRNA‐Seq) data coupled with spatial transcriptomics data.

**Method:**

We performed cell‐cell interaction analysis of astrocytes and microglia with L2/3 neurons using LIANA—an open‐source ligand‐receptor analysis framework—on a snRNA‐Seq dataset from the inferior temporal gyrus (ITG) region of 32 donors spanning the entire continuum from normal aging to severe AD neuropathology. We identified ligands‐receptor pairs that were specific to L2/3 neuron interactions with astrocytes and microglia, and were correlated with pTau and/or L2/3 neuronal loss. We performed a preliminary analysis of the specificity of these interactions in L2/3 with spatial transcriptomics on an AD donor.

**Results:**

The cell‐cell interaction results indicated that L2/3 neurons interacted most with microglia subclusters mic.1, which is enriched in antigen presentation, complement, and ribosomal genes and mic.7, which is enriched in cytokine response (Figure 1). Notably, *APOE* expression in mic.1 was correlated positively with pTau and negatively with L2/3 neuron proportion. The *APOE* receptor gene *LRP1* was also positively correlated with pTau and negatively correlated with L2/3 proportion in L2/3 neurons. Moreover, *APOE* expression was higher in cells near L2/3 and L5 neurons relative to other cortical layers and further analysis suggested enrichment of mic.1 in layer 2/3 (Figure 2). The cell‐cell interaction analysis also revealed specific interactions of L2/3 neurons with homeostatic and reactive astrocyte subclusters. Among those, *SEMA3D* was positively correlated with pTau in all astrocyte subclusters and its receptor *PLXND1* was also positively correlated with pTau and negatively correlated with L2/3 proportion. However, *SEMA3D* expression was not specific to layer 2/3.

**Conclusion:**

Our integrative analysis of snRNA‐Seq and spatial transcriptomics data highlighted critical ligand‐receptor interactions with microglia and astrocyte subclusters that are associated with L2/3 neuron loss in AD. These findings underscore the complex interplay between neuronal and non‐neuronal cells in AD‐related neurodegeneration.